# A218 THE RISK OF RECURRENT HEPATOCELLULAR CARDINOMA IN POST-LIVER TRANSPLANT PATIENTS RECEIVING CAPECITABINE TREATMENT

**DOI:** 10.1093/jcag/gwab049.217

**Published:** 2022-02-21

**Authors:** M Alsager, V Chaitanya, R Gandhi, K Puka, E Tang, A Teriaky, K Qumosani, A Skaro, J Parfitt, M Brahmania

**Affiliations:** Department of Medicine, Western Univesity, London, ON, Canada

## Abstract

**Background:**

Little is known on how to reduce the risk of hepatocellular carcinoma (HCC) recurrence post liver transplantation (LT). We examined if adjuvant oral Capecitabine reduces the risk of recurrent HCC in a high-risk group post-LT.

**Aims:**

To examine if adjuvant oral Capecitabine reduces the risk of recurrent HCC in a high-risk group post-LT.

**Methods:**

A retrospective study was performed from a pre-existing liver transplant database from the Liver Transplant Unit at London Health Sciences Center, London; Canada. This database contains demographic, clinical parameters and follow-up of all patients transplanted for HCC. Data was extracted for patients who underwent LT between January 2000 – April 2018 and included follow up until May 31^st^, 2020. High-risk of tumor recurrence was defined as a RETREAT score ≥5 or PARFITT score ≥10.5. Log rank test compared the recurrence of HCC or death among patients who were and were not prescribed Capecitabine.

**Results:**

Out of 168 LT for HCC, 25 patients were identified as high-risk group for recurrence. The median age was 63 years (IQR=60–65). 19 (76%) patients had viral hepatitis including Hepatitis B and Hepatitis C as their primary disease while 4 (16%) patients had NASH. The remaining 2 (8%) patients had Autoimmune Hepatitis. 7 (28%) patients received Capecitabine while 18 (72%) did not. All patients were followed for a median of 22 months (IQR=8.9–57.5). No statistical significance difference was found between the two groups with respect to HCC recurrence or death (p=0.56).

**Conclusions:**

Among patients with high risk features for recurrence of HCC, adding Capecitabine therapy added to conventional immunosuppression had no overall effect on reducing overall tumor recurrence or survival.

**Funding Agencies:**

None

